# The interval since first symptoms until diagnosis of squamous cell carcinoma in the head and neck region is still a problem in southern Brazil

**DOI:** 10.4317/medoral.23781

**Published:** 2020-10-09

**Authors:** Larissa Balbo Zavarez, Roberta Targa Stramandinoli-Zanicotti, Laurindo Moacir Sassi, Gyl Henrique Albrecht Ramos, Juliana Lucena Schussel, Cassius Carvalho Torres-Pereira

**Affiliations:** 1 Postgraduate Program in Dentistry, Federal University of Parana (UFPR), Curitiba, PR, Brazil; 2 Department of Oral and Maxillofacial Surgery, Erasto Gaertner Hospital, Curitiba, PR, Brazil; 3 Department of Head and Neck Surgery, Erasto Gaertner Hospital, Curitiba, PR, Brazil; 4 Associate Professor at the Department of Stomatology - Federal University of Parana (UFPR), Curitiba, Brazil

## Abstract

**Background:**

The aim was to examine the interval since first symptoms until final diagnosis of squamous cell carcinoma (SCC) in the head and neck region in southern Brazil.

**Material and Methods:**

The individuals were prospectively selected and underwent anamnesis, physical examination and interview in the first medical consultation at a Cancer Hospital from south of Brazil.

**Results:**

From 488 patients who underwent clinical examination, 105 were included in the study with diagnosis of SCC. Patients average interval from first symptoms to final diagnosis was 152 days (median 86; max:1105; min: 1), the average professional interval was 108 days (median: 97; max:525; min: 1) , and the average total period interval was 258 days (median: 186; max:1177; min: 45). Factors statistically associated with patient and diagnosis itinerary intervals were smoking and poorly adapted dentures and distance from home to hospital, respectively.

**Conclusions:**

The identification of the itinerary characteristics of this specific population may reflect in more effective public policies, such as primary and secondary prevention programs, aiming to increase the survival of oncological patient. Furthermore, the knowledge of the variables that influence the late diagnosis minimizes patient's journey in search of care to cancer centers through health programs.

** Key words:**Head and neck cancer, time interval, time to diagnosis, diagnosis delay, squamous cell carcinoma.

## Introduction

Most head and neck tumours are diagnosed in advanced stages as stage III or IV, mostly associated to delays in the diagnosis. Tumour stage at diagnosis is recognized as an important prognostic marker for differents types of cancer. When the interval from symptom presentation to initial appointment at primary care exceeds one month, the chances of advanced-stage tumour at diagnosis is significantly higher (1,2). Therefore, cancer diagnostic interval length is considered an important risk factor for mortality in head and neck carcinomas, which is a public health priority, especially in the underdeveloped countries, with almost 130,000 annual deaths worldwide (3-7). Tumour stage at diagnosis is recognized as an important prognostic marker for differents types of cancer (8).

Historically, differents criteria have been used to classify the periods before the treatment. Most studies have defined the patient interval as the period between the patient first sign or symptom noticing and their first consultation with a healthcare professional. The professional interval have been described as the time elapsed since the patients first consultation with a healthcare professional to the definitive pathological diagnosis or until the appointment for treatment (1,8,9).

The definitions of the “Aarhus Statement” recommended the term “patient delay” should no longer be used, using instead the term “evaluation interval” which is time taken to interpret bodily changes/symptoms, and “help-seeking interval” which is time taken to act upon those interpretations and seek help, should be more precisely helpful in describing the “patient interval” (10).

There are many examples in the literature, which consider both the patient and the professional or just one of them at the diagnostic interval in cancer. Howsoever, this study is an attempt to summarise factors about the actual contribution of each of these time intervals for patients to follow towards their definitive diagnosis and treatment. This information may play a key role in prioritizing early diagnosis interventions in head and neck cancer, through either community-based or primary care level in the healthcare system. Therefore, the aim of this study was to examine the length period of evaluation interval, as well as the length of the help-seeking interval in the head and neck region cancer individuals.

## Material and Methods

This was a cross-sectional study based on the data collection through interview and evaluation of medical records.

The study included individuals aged 18 years or above, that had their first medical consultation at the Hospital between the period August, 2016 to April, 2017, who presented oral lesions with suspicion of malignancy.

The interviews were conducted by a single researcher (LBZ). All patients answered the questionnaire voluntarily, but only the individuals who had the final diagnostic of oral or oropharyngeal squamous cell carcinoma were included in the study. The confirmation was given by histopathological analysis after biopsy (International Classification of Diseases, 9th and 10th revision, C 00 to C14). Patients with secondary tumours or other previous cancer treatment were excluded from the sample.

Anatomic locations were divided into two sites: mouth and oropharynx. Mouth included tongue, gums, floor of the mouth, buccal mucosa, hard palate and lips. The oropharynx tumours were considered when extending from the uvula to the level of the hyoid bone.

The questionnaire covered demographic data, harmful habits related to oral and oropharyngeal cancer, and medical history. The evaluation interval was registered as a continuous variable measured in days and also divided into separate categories in periods of months (11) from the date when the patient reported the beginning of the signs or symptoms until the time of diagnosis in the specialized cancer service. In order to calculate the time of evaluation interval in seeking professional help, participants were asked to state a date when mouth symptoms were first noticed by themselves, and the date they first sought help for those symptoms. Information about clinical characteristics, tumour site and its extension, and symptoms characteristics were also assessed. The patient interval was dichotomized with the cut-off points of “less than one month” (no delay) and “more than one month (delay) (12).Therefore we classified as "delay" any time greater than thirty days (11-13) since patients that present a potentially malignant mouth lesion or symptoms for over 2 or 3 weeks should be advised to seek a healthcare professional. The help-seeking intervals were evaluated as a continuous variable measured in days and also divided into separate categories in period of months (11).

Based on the literature (10), we have defined that the "evaluation interval" is from the onset of the first symptom to the first investigation by the patient's primary care provider. The "help-seeking interval" corresponds the period between the first investigation date and the final diagnosis date. So the "total interval" is the total time between first symptom and the date of the pathology report. Therefore, for "total diagnosis interval" concept we have used the period between the patient first noticing sign or symptom and the ultimate diagnosis. As it is described in the literature, these terms are also related to the “patient delay” and “professional delay” being like, now related patient interval and doctor interval with system interval respectively, which we will choose to call all this dependent period of the health professional and of the health system as professional interval also called the diagnostic interval (10).

The variable “total interval” were correlated with the following independent variables: tumour site, gender, skin color, age, distance from health tertiary care, years of study, income, smoking, alcohol intake, UV exposure, poorly adapted denture, pain, tumour staging, symptom and dental attendance. Individuals were divided into two groups: smokers vs. non-smokers and non-drinkers vs. drinkers, respectively. Patients claiming to have quit smoking or alcohol drinking have been classified as “former users”. Dental attendance was defined as regular when the subjects claimed to have undergone dental visit at least once a year.

 The information collected was transferred to a database and a descriptive analysis was carried out with a presentation of the measures of central tendency and dispersion for continuous variables and number and percentage of category variables. All analyses were done with SPSS statistical software (version 10.0), and 95% confidence intervals were estimated by logistic regression analysis. The Fisher Exact Test was applied to analyze the dichotomous qualitative variables and the Chi-Square Test to analyze the other categorical variables. The value of *p*<0.05 was considered statistically significant.

## Results

Four hundred eighty-eight patients were admitted in the Department of Head and Neck Surgery during the study period and answered the questionnaire. The diagnosis of squamous cell carcinoma occurred in 21.5% of patients (n=105). Patients were excluded when the following criteria were met: diagnosis of benign tumour (n=4, 0.8%), patient with previous tumour treatment (n=13, 2.7%), past history of malignant disease or previous history of cancer (n=36, 7.4%), loss of treatment follow-up (n=47, 9.6%), presence of another carcinoma (n=58, 11.9%), other tumour sites (n=225, 46.1%). Therefore, the analyses were based on a sample of 105 participants - 53 patients with oral squamous cell carcinoma (50.5%) and 52 patients with oropharyngeal cancer (49.5%). The age when mouth sign/symptoms were detected ranged from 20 to 87 years (average of 60.4y). Most of the patients were men (n=88, 84%) and 17 (16%) were women. Ninety-three individuals (89%) were Caucasian. The epidemiologic profile of the subjects considering elapsed time for healthcare attention is shown in Table 1.

**Table 1:**
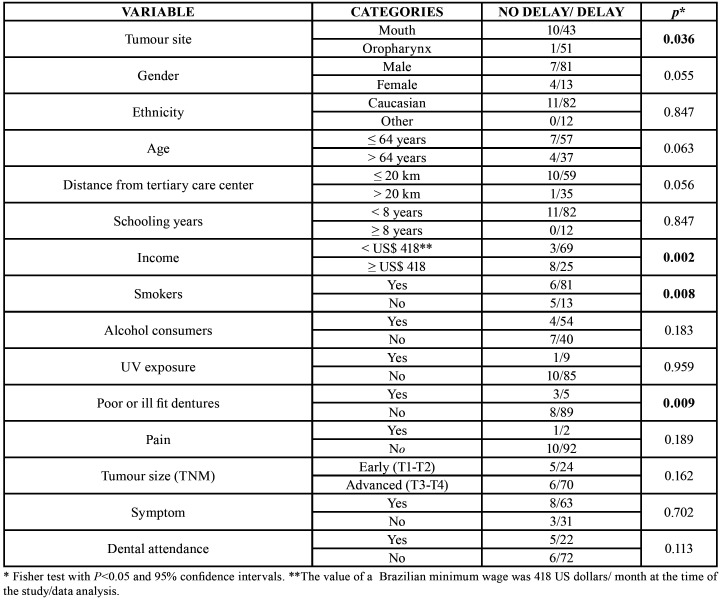
The epidemiologic profile of the subjects considering elapsed time for healthcare attention.

The anatomical distribution of the primary cancer tumours were: tongue (n=43, 40.9%), lips (n=16, 15.2%), oropharynx wall (n=14, 13.3%), hard palate (n=12, 11.4%), buccal mucosa (n=8, 7.6%), soft palate (n=6, 5.7%), floor of the mouth (n=4, 3.8%) and gingiva (n=2, 1.9%). The distribution of patient interval by the site of primary cancer tumour is shown in Table 2. Twelve individuals (11.5%) presented at primary care within 1 month from the reported first symptoms. Fifty-one (48.5%) presented ranging from 1 month to 3 months and 25 (24%) with an interval ranging from 3 to 6 months. A number of 17 (16%) subjects presented at primary care with more than 6 months from first perceived symptoms.

Ninety-three patients (88.6%) were smokers and 12 (11.4%) had never smoked cigarettes. Fifty-eight patients (55.2%) reported daily alcohol consumption, whereas 47 (44.8%) did not. The average patient interval period was calculated based on 105 patients with oral cavity and oropharyngeal cancer and it was 152 days (maximum= 1105 days and minimum 1 day), the average professional interval was 108 days (maximum= 525 days and minimum 1 day), and the average total period interval was 258 days (maximum= 1177 days and minimum 45 days). The individuals distribution according to the categorized intervals is listed in Table 3.

Variables such as gender, age, educational level, skin color, alcohol consumption, family history of cancer, and sun exposure were not related to the dichotomous intervals analyzed (<30 days x >30 days). Regarding to socioeconomic status, 95.8% of the patients earning a Brazilian minimum wage were less likely to search for healthcare when compared to the ones with higher incomes (*p*=0.004). Regarding to smoking habit, 93.1% of smokers took longer time to seek healthcare compared to patients who did not smoke (*p*=0.038). Only 7.6% (n=8) of patients presented poor fitting dentures, of which 62.5% (n=5) took more time to seek healthcare when compared to individuals who did not use complete dentures (*p*=0.036). All of the subjects with poor fitting dentures took a greater time from initial consultations until the definitive diagnosis (professional interval) (*p*=0.031). Considering clinical staging, both patient interval (*p*=0.034) and total interval (*p*=0.037), measured in days, were higher as more advanced was the tumour classification.

Patients who do not go to the dentist regularly took longer to search for healthcare than patients who reported visiting the dentist twice a year (*p*=0.037). The average time of professional interval was 108 days, ranging from the maximum time of 525 days in a case of a 69-year-old male, diagnosed with a lesion on the hard palate; and a minimum of 1 day in a case of a 58-year-old male with a tongue cancer.

**Table 2:**
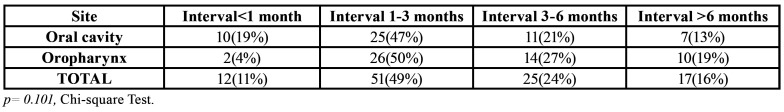
Distribution of patient interval by the site of primary cancer.

**Table 3:**
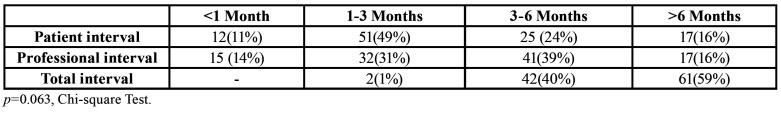
Number of patients according to intervals.

## Discussion

The present study reinforces oral and oropharyngeal cancer high levels of patient and professional interval in patient itinerary from initial cancer presentation to the definitive diagnosis using parameters described in the Aarhus statement. The enormous diagnostic interval length is still a problem worldwide and also in the Brazilian Public Health System.

The Aarhus statement intention is to improve design and reporting of studies on early cancer diagnosis. Moreover, information about primary care and diagnostic intervals have been obtained from the interview and medical records, which increases data reliability and minimizes recall bias when compared to exclusively retrospective studies. It also reduces participant's memory bias, one used the Landmarking calendar (14) adapted as data and national holidays. In order to minimize memory bias yet, particularly those related to the date of first symptoms detection and date of first presentation, patient self-reported information was checked against patient’s relatives and clinical records both at primary care and hospital levels.

There is abundant evidence that early diagnosis would reduce the morbidity and mortality from oral cancer (1,2). The early diagnosis should be easy to achieve, as the oral cavity is easy to explore, but about six of ten oral cancer cases are diagnosed at advanced stages resulting in low survival rates. A pilot study in Spain revealed a low awareness of oral cancer, and a poor knowledge of its signs and symptoms and risk factors (15).

Patients with delayed diagnosis have significantly higher probability to present an advanced-stage tumour at diagnosis than patients with no delay in diagnosis similarly to previous studies (16-18). It may be possible that the relationship between delay in diagnosis and advanced oral and oropharyngeal tumour stage is explained by the fact that certain cancers remain silent during the initial stages and produce symptoms only when in advanced phases. The symptom may not have been uncomforTable or serious enough to request professional consultation. Additionally, patients’ misinterpretation of lesions as benign or self-limiting could considerably influence the patient's interval (12,19).

A quantitative systematic review about the relative length of the patient and primary care intervals in symptomatic oral cancer showed that patient interval represents the major component of waiting times since the detection of the first signs/symptoms to the definitive diagnosis of oral cancer. For the authors, strategies focused on high-risk patients should be prioritised, as interventions aimed at optimising the health systems (20).

Some studies suggest that approximately 30% of patients with oral cancers seek professional help more than 3 months after presenting first symptoms (12,18). These results are similar to the present study that showed 34% individuals were diagnosed with mouth cancer and 46% for oropharyngeal cancer in that patient interval. The patient interval can be explained by different factors such as initial interpretation of symptoms, self-knowledge of mouth anatomy and common aspect, severity of life events in the patients’ interval, deprivation, and the perceived ability to seek help for the symptom (4,12,21). As long as the symptoms of mouth cancer and of oropharyngeal cancer are not specific, it is the professional, not the patients themselves, who are expected to be able to suspect for concerning lesions. Also, the nature of the symptom is not always the driving factor behind help-seeking. The circumstances in which the symptoms presents and the individual’s beliefs about obtaining help may play an important role in the decision to seek help (11). To minimize the first step, interval attributed to patients, many authors state the importance of patient education and recommend a regular examination by a professional. However, there is no data confirming if periodic professional examinations and regular dental care could shorten the cancer delay diagnosis attribuTable to patient interval (11,16).

In this study, it was noticed that patients whose dentures did not fit properly showed a tendency for longer period in professional interval, results also found by other authors (22). This results should be taken with caution, since only eight of the present sample were considered users of an ill fitting denture. Professional interval may be associated with factors such as professional lack of knowledge and/or the lack of preparation of general dental or medical staff (23) in relation to suspicious or malignant lesions, what make it difficult to refer patients. Access to health services, appointments, usage of public transportation, cultural and social backgrounds maybe additional explanations for a larger interval (5,7). Although some studies (8,24-26) report that patient interval is the most significant contributor to delay in the diagnosis, in our study both patient and professional compromised the total interval. Interestingly, some authors have described lower professional intervals in advanced malignancies that should be interpreted as a bias of urgent care and taken into account for future studies regarding the interval from diagnosis to treatment. (20,27-29).

A significant higher patient interval in seeking the advice of a health professional after self-discovery of oral and oropharynx cancer symptoms is a major endpoint for current empirical research to provide clear reasons for this delay. Data obtained from descriptive studies are essential to prioritize interventions in prevention initiatives to promote early diagnosis for mouth cancer. In order to reduce overall diagnostic interval, strategies must include political actions that assure a reduction in the time patient takes to see a healthcare professional coverage, the optimization of the primary care services, specific educational measures focused in giving selective access and priority to patients at high risk or with signs or symptoms of cancer (8,30).

Therefore, the identification of the socio-demographic characteristics of a specific population seems to reflect in public policies for the control of mouth and oropharyngeal cancer, such as the development of a primary prevention program for squamous cell carcinoma. Prevention strategies should be prioritized to educate patients to seek professional help as soon as the first signs and symptoms are observed, since when diagnosed in the early stage, oral and oropharyngeal carcinomas have better healing and survival conditions.

## Conclusions

There is still an important total interval from the first symptoms and diagnosis of oral and oropharyngeal cancer in southern Brazil. A higher interval was mostly related to male gender, oropharynx location, higher distance from tertiary care center, lower income, being an actual smoker and using poor fit dentures. Medical and dental staff should be aware of such factors in order to prioritize and give special attention to the barriers that could be negatively impacting the time elapsed from oral and oropharyngeal cancer symptoms until treatment. With the knowledge of the variables that influence the late diagnosis, it could be possible to minimize patient's journey in search of care to cancer centers through health programs aimed to this population thus reducing morbidity and improving survival.
